# Supplementary material for: AiiDA 1.0, a scalable computational infrastructure for automated reproducible workflows and data provenance
Source: Sci Data. 2020 Sep 8;7:300. doi: 10.1038/s41597-020-00638-4 (PMC7479590; doi:10.1038/s41597-020-00638-4)
Supplement: Supplementary file 1 — Supplementary Information [file 41597_2020_638_MOESM1_ESM.pdf]

Supplementary Information: AiiDA 1.0, a scalable computational infrastructure for automated reproducible workflows and data provenance

A. Architecture differences with earlier AiiDA versions

While the ADES model and the overall goals of AiiDA have remained the same as those originally published in Ref. [S1], AiiDA now comes with many new features over the 0.x series, with the first public release in Feb 2015 and including 9 major releases and 20 releases in total (aiida.net/download); most of the code has been completely redesigned. Improvements aimed towards making AiiDA scalable, and able to support high-throughput computational loads consisting of high-performance computing (HPC) jobs of various size, making it more flexible by simplifying the protocols by which it can be extended, and providing tools and interfaces to facilitate its use. A schematic overview of the architecture is shown in Fig. 1 of the main paper.

The engine, the component responsible for running all calculations and workflows, is described in detail in "The engine" section of the main paper. In a nutshell, the switch from a polling-based design to an event-based one allows the engine to react instantaneously to state changes, making it much more responsive. In addition, the new engine is made scalable by allowing an arbitrary number of independent workers to operate in parallel, supporting now sustained throughputs of more than tens of thousands of processes per hour. Communication between and with the workers is provided by the RabbitMQ message broker (rabbitmq.com) through the pika client library (pypi.org/project/pika).

In addition to increased efficiency, the engine has also been made more robust and now comes with built-in error handling for transient problems, such as connection issues or computational clusters going offline unexpectedly. Failed executions are automatically rescheduled and, if repeated consecutive failures occur, the calculations are paused such that the problem can be investigated by the user. The new engine also comes with improvements in terms of usability. The new workflow language is much more expressive and enables the writing of auto-documenting and reusable workflows.

The two different concepts of calculations and workflows, which behaved and were implemented differently in earlier versions of AiiDA, have now been fully integrated with a homogenised interface, while the entire provenance graph is still stored in PostgreSQL (postgresql.org), a Relational Database Management System (RDMS). As part of the integration of calculations and workflows, the latter are now also fully part of the provenance graph, with the links between them explicitly represented. For this reason the ontology of the AiiDA provenance graph has been revisited and rigorously defined as described in "The provenance model" section of the main paper. The extended provenance graph provides more valuable information to the user, while giving them full control over the level of detail with which to inspect it. To facilitate efficient queries of data and provenance, a dedicated tool has been developed, the `QueryBuilder`, allowing users to write advanced graph queries directly in the familiar Python syntax. The queries are translated to SQL using SQLAlchemy (sqlalchemy.org), a powerful object-relational mapping (ORM) library, which has also been used as a fully-fledged ORM implementation in addition to the original one using Django (djangoproject.com). To allow different ORMs in AiiDA, the original implementation, initially tightly coupled to Django, was decoupled and an abstract AiiDA frontend ORM was constructed. This new ORM interface provides a stable API for the user, independent of the backend implementation and makes it easy to implement other backend solutions.

A completely new way of accessing the AiiDA provenance graph is provided by a REST API server (see "The REST API" section in the main paper) that allows one to retrieve data over HTTP(S) requests. This new component enables users to browse the provenance graph programmatically, and implement custom interfaces like the graphical one provided by the Materials Cloud [S2] web platform.

One of the original methods of interacting with AiiDA, the command line interface (CLI) `verdi` has been significantly improved. The management of command-line input has been replaced with the mature `Click` (click.palletsprojects.com) library. This guarantees an interface that behaves consistently across all commands and makes it easy to reuse components for additional CLIs that can be distributed through plugins.

Finally, plugins to extend AiiDA's core functionality are now easier to write, share and install thanks to a new flexible plugin system (see "The plugin system" section in the main paper). Although the original version of AiiDA already allowed its functionality to be extended through plugins, the code had to be placed in the source tree of AiiDA itself, tightly coupling the development cycle of the core code and of the plugins. The new system allows plugins to be developed independently and to be installed with a single command. A plugin registry (aiidateam.github.io/aiida-registry/) has been deployed, serving as a central place where users can discover existing plugins.

The combination of all these changes, which are discussed in detail in the following sections, aim to make AiiDA 1.0 into a powerful and flexible workflow and data management system ready to manage high-throughput HPC jobs on future exascale machines.

B. Engine details

This section provides some more detail on the internal design and functionality of the engine (for a complete description, see Ref [S3]).

1. *Process runners and the AiiDA daemon*

When a process is launched, an instance of the relevant `Process` subclass is created and assigned to a `process runner` (or simply runner), which runs it to completion. Each runner has an internal event loop, which allows it to run multiple AiiDA processes concurrently in a single thread using coroutines (i.e., functions that can yield control during execution when they need to wait for some long-running action to happen; the event loop can then give control to other coroutines that had previously yielded and are ready to continue). Additionally, each runner has access to a `persister`, which implements the logic to serialise and store the state of a process as a checkpoint (in the specific implementation of AiiDA, by serialising the process instance to YAML format and storing it in the database as an attribute of the corresponding node). This mechanism allows interrupted processes to be restarted, eliminating the need to re-execute code that was already run even if the runner is completely stopped.

The simplest implementation of the runner is the local one, meaning that the process is executed in the current Python interpreter and blocks it until all processes have finished. However, this approach does not scale well for large numbers of processes. AiiDA therefore implements daemon runners, that operate exactly like local runners, except that they are spawned in a separate system process that is ran in the background. We stress here the difference between an AiiDA process, i.e., an entity specific to AiiDA that has data as inputs and as outputs; and a system process, e.g., a Windows or Unix process, that represents the execution of an executable in a computer operating system. Each daemon runner is fully independent from the others and special care has been devoted in the implementation to allow to run multiple runners in parallel without concurrency issues when accessing the database. Given that the daemon runners operate in separate system processes, a message broker is employed to allow communication with and among the runners, as discussed in the next section.

Daemon runners are spawned and controlled by a single main daemonised process, implemented by the circus library (circus.readthedocs.io). AiiDA's `verdi` command line interface provides easy commands to start and stop the daemon, inspect the status of the daemon runners it controls, and to increase or reduce the number of active runners.

2. *Process communication via the RabbitMQ message broker*

Communication with the runners and among runners is provided by the RabbitMQ message broker (rabbitmq.com). Each daemon runner subscribes to a special task queue on the message broker to which newly submitted processes are added as `tasks`. The contents of the task queue are persisted to disk by the broker, so that even after an interruption (for example a machine restart), uncompleted tasks can be reloaded and resent to the runners. The combination of queue persistence, together with the automatic checkpointing of AiiDA processes performed by the engine, ensures that interrupted processes can continue from the last checkpoint, reducing the loss of computational time to the minimum.

The implementation of RabbitMQ and the configuration of the task queue guarantee that each task is only sent to exactly one runner at a time and will eventually be executed. A task is kept in the queue until it is explicitly acknowledged as completed by the runner that received it. The broker monitors subscribed runners by sending a periodic "heartbeat" call. If the runner fails to respond within a certain interval twice consecutively, the runner is considered unreachable and tasks assigned to it are redistributed. To prevent runners from missing the heartbeat call while the main thread is under heavy load, all communication with the broker is performed on a separate thread. In order to avoid race conditions in database updates, the communication thread has no access to the database but only schedules callbacks on the event loop of the main thread, and forwards broadcasts to the broker.

When a runner receives a task to run a process, it subscribes to a dedicated channel for that specific process. In such a way, interaction with live processes (e.g., to pause, restart or kill them) is possible by sending remote procedure calls (RPCs) over the appropriate channel. Conversely, processes can also emit state changes through their runner's communicator. These are broadcast to subscribed listeners (e.g., a parent workflow waiting for all of its subworkflows to finish) that can then respond with an appropriate action. This event-based model makes the AiiDA engine able to reply almost instantaneously to events, without the need to periodically poll and check the process states, which would be much more CPU-intensive and less reactive.

C. Query builder syntax example

To provide a concrete example of the query builder syntax, as implemented in AiiDA's `QueryBuilder` class, we show in Fig. S1 a sample query together with a graphical representation of its action and result. In this example, we query for calculations run with a specific code to compute the total energy of a crystal structure after having relaxed it to within a certain threshold. The goal is to

```
1  qb = QueryBuilder()
2  qb.append(CalcJobNode,
3    tag='calculation')
4  qb.append(Code,
5    filters={'label': 'my-code'},
6    with_outgoing='calculation')
7  qb.append(Dict,
8    with_outgoing='calculation',
9    filters={'attributes.type': 'relax'},
10   project=['attributes.threshold'])
11 qb.append(Dict,
12   with_incoming='calculation',
13   edge_filters={'label': 'results'},
14   project=['attributes.energy'])
```

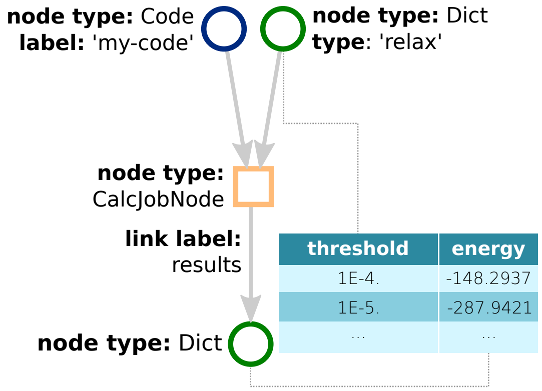

FIG. S1: Top: a query builder graph query to filter all calculations that computed the energy of a structure after relaxing it to within a certain threshold, using a code labeled `my-code`. The result will be a pair of values for each matching result, containing the relaxation threshold (`threshold`) and the computed total energy (`energy`), retrieved from the attributes of the appropriate nodes. The details of the query syntax are explained in the main text. Bottom: graphical representation of the same query.

obtain the total energy of the structure as a function of the relaxation threshold.

To run a new query, first a new query object `qb` is created (line 1). Nodes to be matched are specified by using the `append` method of the query object, in which also additional filters can be declared together with the relation to the other nodes in the query. Finally, "projections" indicate which specific properties of the node should be returned as query results.

To perform the query, we first instruct that we are looking for `CalcJobNode`s (lines 2-3) that we tag as `calculation` to be able to refer to it later when defining inter-node relationships. The calculation should have a code (line 4) with label `my-code` (line 5) as an input (line 6). Additionally, the calculation should have a `Dict` input (line 7-8) containing an attribute `type` with value `relax` (line 9). Instead of the entire input dictionary, we request only the value of the `threshold` attribute to be returned (line 10). Finally, the calculation needs to have an output `Dict` node (lines 11-14) connected by a link with label `results` (line 13), and we project the `energy` attribute (line 14).

To evaluate the query, one can then simply run the method `qb.all()` that returns a list of results, one for each subgraph matching the query. Each result is an ordered list of the values that were defined by the projections, in this case the two values (`threshold`, `energy`). The final query result then has the form `[(threshold1, energy1), (threshold2, energy2), ...]`.

D. Example of a work chain and calculation function

To showcase the interface of AiiDA's workflow language described in the "The engine" section of the main paper, we implement a simple Fibonacci number calculator. The Fibonacci sequence is defined as:

$$f_N = f_{N-1} + f_{N-2} \tag{S1}$$

where $f_0 = 0$ and $f_1 = 1$. Fig. S2(a) shows a possible implementation using a work chain and a calculation function. The `outline` codifies the logical sequence of the work chain. The first step `initialize` will set some context variables, such as an `iteration` counter and the initial Fibonacci numbers f_0 and f_1, which correspond to `previous` and `current`, respectively. The `ctx` member of the work chain is a `context` that is persisted between the logical steps and can be used to transfer information between them. The `while_` logical construct is used to tell the workflow to iterate until $N-1$ iterations have been performed. Each iteration consists of summing the integers in the `previous` and `current` variables, which directly corresponds to Eq. (S1). For this addition the `add` calculation function is called such that the provenance is kept. Finally, after N iterations, the `current` value is returned as the requested Fibonacci `number` in the `results` step.

Fig. S2(b) shows the provenance graph that is produced by AiiDA when running the `Fibonacci` work chain. Note that not only the work chain with the initial input and the final answer is represented, but also all individual additions performed along the way, with their intermediate results. This implementation of a Fibonacci sequence calculator, while clearly overengineered, shows how arbitrary logic can be implemented in AiiDA's workflow language and how provenance is automatically stored.

[S1] G. Pizzi, A. Cepellotti, R. Sabatini, N. Marzari, and B. Kozinsky, Computational Materials Science **111**, 218 (2016).

```
@calcfunction
def add(x, y):
  return x + y

class Fibonacci(WorkChain):

  @classmethod
  def define(cls, spec):
    super(Fibonacci, cls).define(spec)
    spec.input('N', valid_type=orm.Int,
      help='Compute the Nth Fibonacci number.'
    )
    spec.outline(
      cls.initialize,
      while_(cls.should_iterate)(
        cls.iterate),
      cls.results)
    spec.output('number', valid_type=orm.Int)

  def initialize(self):
    self.ctx.iteration = 0
    self.ctx.previous = orm.Int(0)
    self.ctx.current = orm.Int(1)

  def should_iterate(self):
    return self.ctx.iteration < self.inputs.N - 1

  def iterate(self):
    previous = self.ctx.current
    self.ctx.current = add(
      self.ctx.previous, self.ctx.current)
    self.ctx.previous = previous
    self.ctx.iteration += 1

  def results(self):
    self.out('number', self.ctx.current)

number = run(Fibonacci, N=orm.Int(5))
```

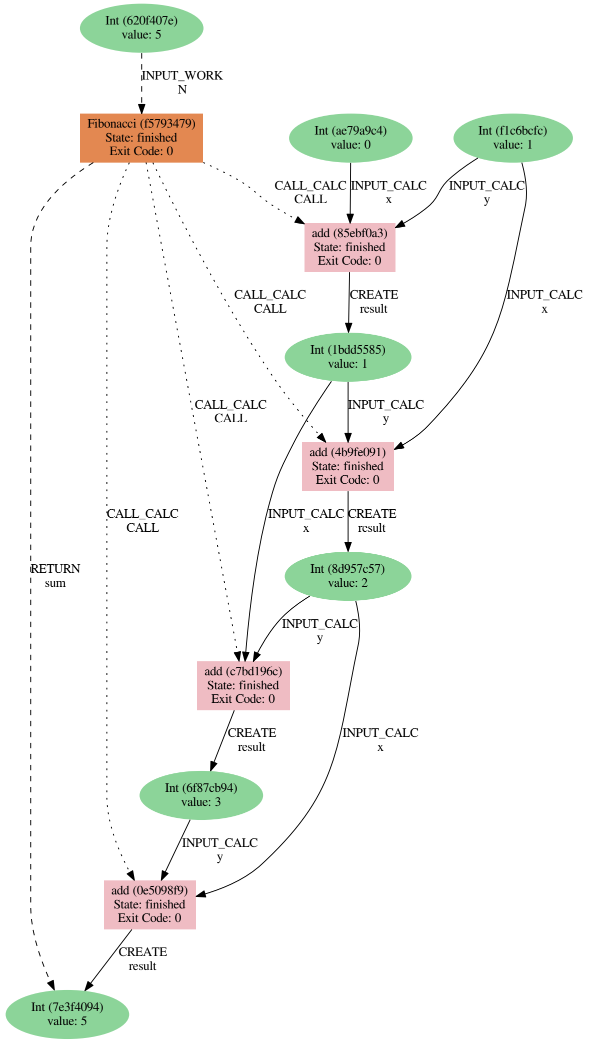

FIG. S2: (a) Example implementation to compute the Nth Fibonacci number using AiiDA's workflow engine. The `Fibonacci` work chain implements Eq. (S1) and leverages the `add` calculation function to perform the additions. (b) Provenance graph of the execution of the `Fibonacci` work chain with $N = 5$ as input, executed with the last line of panel (a) and returning $f_5 = 5$ as a result.

[S2] L. Talirz, S. Kumbhar, E. Passaro, A. V. Yakutovich, V. Granata, F. Gargiulo, M. Borelli, M. Uhrin, S. P. Huber, S. Zoupanos, C. S. Adorf, C. W. Andersen, O. Schütt, C. A. Pignedoli, D. Passerone, J. VandeVondele, T. C. Schulthess, B. Smit, G. Pizzi, and N. Marzari, in preparation (2020).

[S3] M. Uhrin, S. P. Huber, J. Yu, N. Marzari, and G. Pizzi, ArXiv e-prints (2020), 2007.10312.
